# A new dataset on plant occurrences on small islands, including species abundances and functional traits across different spatial scales

**DOI:** 10.3897/BDJ.8.e55275

**Published:** 2020-09-10

**Authors:** Julian Schrader, Soetjipto Moeljono, Junus Tambing, Cornelia Sattler, Holger Kreft

**Affiliations:** 1 Biodiversity, Macroecology & Biogeography, University of Goettingen, Goettingen, Germany Biodiversity, Macroecology & Biogeography, University of Goettingen Goettingen Germany; 2 Department of Biological Sciences, Macquarie University, Sydney, Australia Department of Biological Sciences, Macquarie University Sydney Australia; 3 Program Pascasarjana, Program Studi Magiater Kehutanan, Universitas Papua, Manokwari, Indonesia Program Pascasarjana, Program Studi Magiater Kehutanan, Universitas Papua Manokwari Indonesia; 4 Balai Penelitian dan Pengembangan Lingkungan Hidup dan Kehutanan, Manokwari, Indonesia Balai Penelitian dan Pengembangan Lingkungan Hidup dan Kehutanan Manokwari Indonesia; 5 Department of Community Ecology, UFZ - Helmholtz Centre for Environmental Research, Halle, Germany Department of Community Ecology, UFZ - Helmholtz Centre for Environmental Research Halle Germany; 6 Centre of Biodiversity and Sustainable Land Use (CBL), University of Goettingen, Goettingen, Germany Centre of Biodiversity and Sustainable Land Use (CBL), University of Goettingen Goettingen Germany

**Keywords:** Raja Ampat archipelago, West Papua, functional island biogeography, species abundance, species richness, plant functional traits, spatial scale

## Abstract

**Background:**

We introduce a new dataset of woody plants on 60 small tropical islands located in the Raja Ampat archipelago in Indonesia. The dataset includes incidence, abundance and functional trait data for 57 species. All islands were sampled using a standardised transect and plot design providing detailed information on plant occurrences at different spatial scales ranging from the local (plot and transect scale) to the island scale. In addition, the dataset includes information on key plant functional traits linked to species dispersal, resource acquisition and competitive strategies. The dataset can be used to address ecological questions connected to the species-area relationship and community assembly processes on small islands and in isolated habitats.

**New information:**

The dataset yields detailed information on plant community structure and links incidence, abundance and functional trait data at different spatial scales. Furthermore, this is the first plant-island dataset for the Raja Ampat archipelago, a remote and poorly studied region, and provides important new information on species occurrences.

## Introduction

Islands are ideal research models to study ecological processes in spatially discrete arenas (Whittaker and Fernández-Palacios 2007). Detailed understanding of island ecology has led to influential theories in biodiversity research, such as the Equilibrium Theory of Island Biogeography (MacArthur and Wilson 1967) or the General Dynamic Model (Whittaker et al. 2008). These theories are based on species richness on islands to discern assembly processes and biodiversity patterns across islands. However, recent advances in island biogeography advocated for incorporating other biodiversity measures to separate the underlying processes of species assembly on islands. These measures include species abundances (Chase et al. 2019), functional traits (Ottaviani et al. 2020, Schrader et al. 2020a) and community structure at different spatial scales (Craven et al. 2019). For instance, incorporating species abundances provides information on ecological mechanisms behind the species-area relationship (Chase et al. 2019). The species-area relationship describes the increase of species richness with island area and is one of the most fundamental patterns in ecology (Rosenzweig 1995). Functional traits characterise morphological, physiological or phenological features of a species and can offer detailed understanding of ecological filtering (Cadotte and Tucker 2017) and ecosystem functioning (Díaz and Cabido 2001). However, open access datasets that include multiple facets of island biodiversity, such as abundance data and functional traits at different spatial scales, remain scarce.

Here, we provide a novel island dataset that features occurrences, abundances and key functional traits of 57 plant species on 60 small tropical islands. Species occurrences were recorded at three different spatial scales ranging from small-scale plot and transect level data to species communities for the whole island. Furthermore, the study area, lying in the western part of the island of New Guinea, is biologically largely uncharted and the dataset can be used to map species occurrences in this biologically rich region.

## General description

### Purpose

The dataset was assembled with the purpose of investigating the underlying processes behind the island species-area relationship, the small-island effect and community assembly on small islands (e.g., Schrader et al. 2019b, Schrader et al. 2019a, Schrader 2020b). The species-area relationship can form a notable exception for small islands, where species richness varies independently or increases at a different rate with area than on larger islands, a pattern termed the small-island effect (Lomolino and Weiser 2001). The ecological mechanisms behind the small-island effect are still poorly understood. To test whether a small-island effect prevails in the study system, we also included islands with no species in the dataset, as these are important for the correct detection of the small-island effect (Wang et al. 2016).

For all islands, we provide information on island area, island perimeter, island distance to the nearest larger landmass, neighbouring landmass proportion around each island, mean soil depth and proportion of leaf litter coverage on each island. The dataset includes species occurrence and abundance information for woody plants with a diameter at breast height ≥2 cm for each island at three different spatial scales. For each plant species, we sampled key functional traits that we measured from samples collected on the islands. Species occurrences are also available in the Global Biodiversity Information Facility database (GBIF; DOI: https://doi.org/10.15468/zjq49b) and the trait data in the TRY database (Kattge et al. 2020).

## Sampling methods

### Study extent

**Figure 1.**
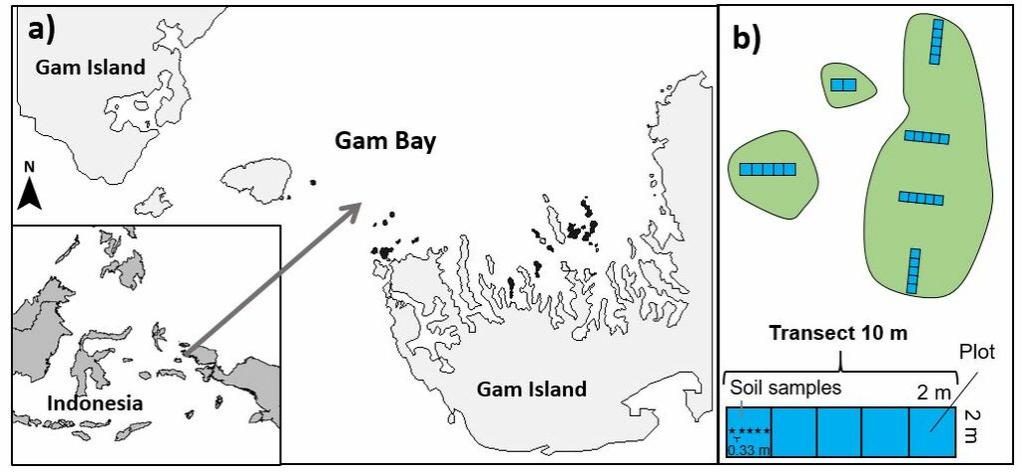
Map of the study region and schematic representation of the study design. a) Location of 60 islands studied in Gam Bay in the Raja Ampat archipelago, Indonesia. The 25 largest sampled islands are highlighted in dark grey. The 35 islands smaller than 100 m^2^ are not visible at this scale. b) Species richness and number of stems were recorded in plots (2 × 2 m) and transects (10 × 2 m). The number of transects placed on an island depended on island area, whereby larger islands received more transects. On islands smaller than the area of a single transect, we placed as many plots as possible.

The dataset includes 60 islands ranging in area size from 3 m^2^ to 11,806 m^2^. All islands included in the dataset are located in the Raja Ampat archipelago in West Papua, Indonesia (Fig. 1). Botanical field surveys and trait sampling were conducted during six months between June 2016 and February 2018. We sampled only islands that were undisturbed by people and covered with woody vegetation, which we ensured by checking for any signs of human use (e.g., clear-cuts, gardens, habitations) or cutting of woody vegetation (see also Schrader et al. 2019a). This excluded all islands that featured gardens, clear-cuts and buildings, limiting maximum island size sampled to <12,000 m^2^, as well as the main island of Gam (Fig. 1).

### Sampling description

Island metrics

We georeferenced all islands in Gam Bay in ArcGis (v.10.3) using satellite images (World Imagery, ESRI 2017). For islands <100 m^2^, we additionally measured the island's dimensions in the field and matched them with the ArcGis georeferenced shapes. Based on the georeferenced shapes, we calculated island area (m^2^) and the perimeter of each island (m). To assess the level of isolation of each island, we calculated two alternative isolation metrics following Weigelt and Kreft (2013). The first isolation metric indicated the minimum distance (m) to the next larger landmass (i.e., calculated as minimum distance from island edge to landmass edge), which was the large island of Gam (Fig. 1). The second metric considered the surrounding landmass proportion within a 1000 m radius around each focal island.

**Plot design**

To sample species occurrences, we used a transect design subdivided into plots (Fig. 1). We used a nested sampling design to obtain information on species assemblages at different spatial scales on the islands (Schrader et al. 2019a). All transects had a dimension of 2 x 10 m and were comprised of five 2 x 2 m plots. The number of transects on an island was roughly proportional to the island area and ranged from one to six transects (one transect was placed on islands <500 m^2^ (40 islands); two transects on islands between 500 m^2^ and 750 m^2^ (two islands); three transects on islands between 750 m^2^ and 1000 m^2^ (two islands); four transects on islands between 1000 m^2^ and 3000 m^2^ (nine islands); five transects on islands between 3000 m^2^ and 5000 m^2^ (three islands); six transects on islands >5000 m^2^ (four islands) (see also Suppl. material 1). For islands with a maximum extension of <10 m we placed as many plots as possible on the island at the longest extension. This was the case for the 36 smallest islands. Larger islands had two transects oriented towards the island centre on the opposite sides of the island. The interior was covered with a varying number of transects (depending on the island size) of perpendicular orientation, ranging from one to four transects. The distance between transects on each island with multiple transects was held constant but was related to the longest extension of an island, and hence varied among islands. With this method we ensured sampling of the island edge as well as the interior, which likely harbour different species communities (Schrader et al. 2019b). Soil depth was recorded in all plots at five spots at equal distance to each other (33 cm) and spaced along the central axis of the transect. At each spot where we measured soil depth, we also recorded the presence or absence of leaf litter.

We recorded all species with a diameter at breast height ≥2 cm rooted within the plots. This allowed us to assess species occurrences at different spatial scales. These scales were i) the plot scale (species sampled in each plot), ii) the transect scale (species sampled along each transect) and iii) the island scale (pooled species occurrences of all transects for each island) (see also Schrader et al. 2019a). For each individual species, we recorded the diameter at breast height in cm (by convention 1.3 m) and the plant height (m). Based on these metrics, we calculated the tree basal area per ha (m^2^ ha^-1^) for each island.

### Quality control

We resolved all taxonomic names using The Plants of the World Online (accessed July 2020). Species were identified with help from local experts and by comparing species samples with vouchers from the Herbarium of the University of Papua. In addition, doubtful species were sent to the Royal Botanical Gardens Kew (UK) for further verification. Seven species were only identified to genus level and nine species could not be identified to species or genus level. For all species, vouchers are deposited in the herbarium of the State University of Papua (UNIPA), Manokwari, Papua Barat, Indonesia. Herbarium IDs for all species are provided in Suppl. material 2.

All plant functional traits were assessed following standardised protocols (Pérez-Harguindeguy et al. 2013). A detailed description of trait sampling methods can be found in the section *Traits coverage*.

## Geographic coverage

### Description

All islands were located in Gam Bay, a large bay of Gam Island, and are sheltered from the open ocean (Fig. 1a). The climate is tropical, mostly calm and lacking pronounced seasonality, with a mean annual temperature of 27.4 °C and annual precipitation of around 2768 mm (weather station Sorong/Jefman; www.worldclimate.com 2020). All islands are composed of coralline limestone, belong to the same limestone plateau and are likely of similar age. Differences in topographic heterogeneity and elevation across islands were small, ranging for elevation between c. one to eight m a.s.l. Mineral soil was absent on all islands. Organic litter, mostly accumulating from dead plant material, was the only basis for soil development on the islands. Stages of decomposition depend on leaf litter depth, which was highly variable, ranging from a few cm to >1 m.

## Taxonomic coverage

### Description

**Figure 2.**
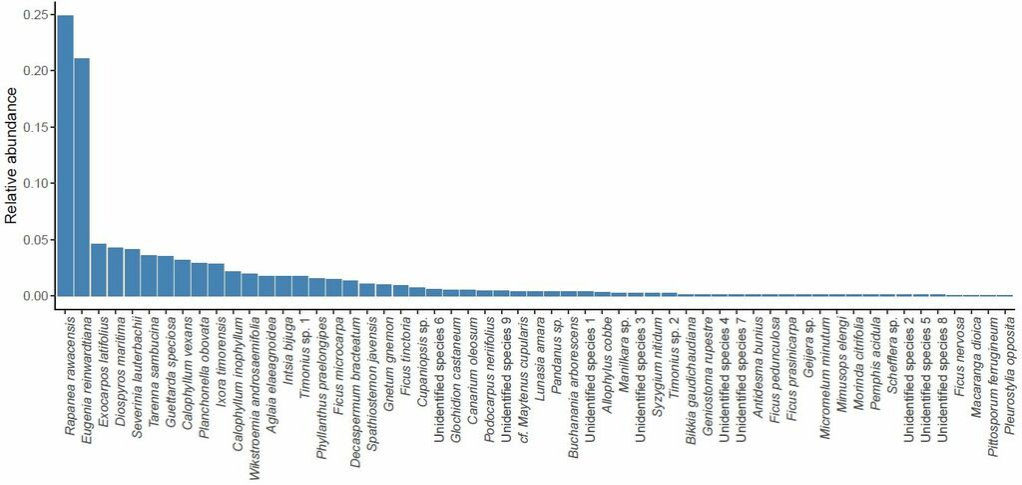
Relative abundance (proportion of individuals) of the 57 woody plant species recorded across all studied islands.

We inventoried all woody plants with a diameter at breast height ≥2 cm (Fig. 2). This included 57 species from 26 families. The most common species were *Rapanea rawacensis* (Primulaceae) and *Eugenia reinwardtiana* (Myrtaceae), accounting for almost 50% of all records. Four species were only recorded once (Fig. 2). All recorded species were native, whereas alien species are not known to occur on the islands (Takeuchi 2003). The community data for all islands and species can be found in Suppl. material 3. Species occurrence data formatted following the Darwin Core standard are also available in Suppl. material 5 and in the Global Biodiversity Information Facility database (GBIF - http://ipt.pensoft.net/resource?r=plant-occurrences_raja-ampat_j-schrader_2020; DOI: https://doi.org/10.15468/zjq49b).

## Traits coverage

We sampled data of ten plant functional traits that cover important dimensions of species life-history strategies (Reich 2014, Díaz et al. 2016, Westoby 1998, Wright et al. 2004): tree height, wood density, leaf area, leaf mass per area (LMA), chlorophyll content, leaf chemical contents (leaf nitrogen, carbon and phosphorous) and seed and fruit mass (Table 1). All traits were measured on individuals growing on the studied islands. Mean trait values for all species can be found in Suppl. material 4. Please note that due to logistical reasons we only measured tree height for each individual (see Suppl. material 3). The mean trait data for all species are also available in the TRY database (Kattge et al. 2020).

We measured tree height of each individual in our dataset using a measuring tape (for individuals <3m) and a measuring stick (for individuals >3m). Tree height for each individual can be found in Suppl. material 3. For species mean tree height, we provided two different measures of tree height. The first measure considered the height of the single largest recorded individual (m). For the second measure, we calculated the maximum tree height (m) as the mean height of the three tallest individuals of each species (following King et al. 2006).

Wood density (g cm^-3^) describes the volume of the main stem divided by its oven-dry weight. Wood samples were dried for 48 h at 100 °C. Branches, bark and green parts were removed prior to measurements. We measured wood density of two mature individuals per species. Including more samples was impossible due to the rarity of many species (Fig. 2).

All leaf traits were measured on ten mature and sun-exposed leaves from several individuals when available. We measured leaf area (cm^2^) using the android application Leaf-IT (Schrader et al. 2017), and leaf dry mass using a digital scale (± 0.001). We oven-dried leaves for 48 h at 80 °C. For leaf mass per area (LMA; g cm^-2^), we divided the leaf area by its dry mass.

For chlorophyll content, we used a chlorophyll meter (Konica Minolta, SPAD – 502DI Plus). We provide the original SPAD units as well as converted the SPAD measurements to chlorophyll concentrations (µm cm^-2^) using the equation by Coste et al. (2010).

Leaf chemical contents (nitrogen, carbon and phosphorous) were measured for the same leaves used for leaf area measurements, by grinding the oven-dried leaves. Leaf nitrogen and carbon concentrations (mg g^-1^) were determined by automated dry combustion (Elementar, Vario EL Cube). Leaf phosphorous concentrations (mg g^-1^) were measured using an inductively coupled plasma-atomic emission spectrometer (iCAP 6300 Duo VIEW ICP Spectrometer, Thermo Fischer Scientific GmbH, Germany).

We collected and measured the dry fruit and seed mass (g) of 44 and 38 species, respectively. We aimed for at least ten fruits per species, which was difficult for some species when fruiting was scarce (the number of fruits sampled per species ranged from 1 to 40; mean = 11.6). Fruit and seeds were oven-dried for 72 h at 80 °C. The fruits of most plants were eaten and dispersed by birds. A checklist of the birds occurring in the study region is provided by Schrader et al. (2020b).

## Usage rights

### Use license

Creative Commons Public Domain Waiver (CC-Zero)

## Data resources

### Data package title

A new dataset on plant occurrences on small islands, including species abundances and functional traits across different spatial scales

### Number of data sets

5

### Data set 1.

#### Data set name

Island data

#### Number of columns

12

#### Description

Data for 60 islands including island coordinates, geo-environmental variables, community summary statistics and number of sampling units. Available as Suppl. material 1.

**Data set 1.** 

Column label	Column description
island_ID	A unique ID for each island.
island_coordinates	Coordinates of each island.
island_area	Total land area of each island.
island_perimeter	Perimeter of each island.
distance_Gam	Shortest distance of each island to the nearest large landmass, which is the island of Gam.
buffer_area_1000m	Neighbouring landmass around each islands within a radius of 1000 m.
tree_basal_area	Tree basal area of each island.
species_number	Species numbers on each island.
soil_depth_mean	Mean soil depth for each island.
leaf_litter_cover	Percentage of leaf litter cover on each island.
no_transects	Number of transects placed on each island. If "0" than only plots were placed on an island.
no_plots	Number of plots placed on each island.

### Data set 2.

#### Data set name

Species data

#### Number of columns

5

#### Description

Taxonomic list of all species found on the studied islands. Available as Suppl. material 2.

**Data set 2.** 

Column label	Column description
species_ID	A unique ID for each species.
Family	Species family
Species	Species name
Author	Species author
UNIPA_Voucher_ID	Specimen voucher ID. Vouchers are deposited in the herbarium of the State University of Papua, Manokwari, Papua Barat, Indonesia.

### Data set 3.

#### Data set name

Community data

#### Number of columns

6

#### Description

Community data for all individuals recorded on the studied islands, including occurrences in transects and plots, diameter at breast height and height. Available as Suppl. material 3.

**Data set 3.** 

Column label	Column description
island_ID	A unique ID for each island. Detailed information for each island can be found in Suppl. material 1.
transect_ID	A unique ID for each transect.
plot_ID	A unique ID for each plot.
species_ID	A unique ID for each species. Scientific names for each species ID can be found in Suppl. material 2.
DBH_cm	Diameter at breast height
tree_height_m	Height of each individual tree

### Data set 4.

#### Data set name

Plant functional trait data

#### Number of columns

13

#### Description

Plant functional trait data for all species. Available as Suppl. material 4.

**Data set 4.** 

Column label	Column description
species_ID	A unique ID for each species. Scientific names for each species ID can be found in Suppl. material 2.
chlorophyll_SPAD	Chlorophyll concentration as measured by a SPAD chlorophyll meter.
chlorophyll_mod	Chlorophyll concentration converted from SPAD units.
fruit_mass	Fruit mass (dry)
seed_mass	Seed mass (dry; average mass for 1000 seeds)
LMA	Leaf mass per area
leaf_area	Area of a leaf
wood_density	Wood density
max_tree_height	Maximal recorded height of each species.
max_tree_height_3	Maximum height of the three tallest individuals of each species.
leaf_N	Leaf nitrogen content in percent.
leaf_C	Leaf carbon content in percent.
leaf_P	Leaf phosphorous content in percent.

### Data set 5.

#### Data set name

Plant occurrences on small islands in the Raja Ampat Archipelago, Indonesia

#### Data format

Darwin Core

#### Number of columns

17

#### Download URL

https://doi.org/10.15468/zjq49b

#### Description

This dataset describes the occurrence of all taxa that are identified at least to the level of genus (nine unidentified taxa are excluded here but can be found in the dataset Suppl. material 2) and can be used as occurrence records and as a taxonomic list for all studied islands. However, the occurrence records cannot be regarded as a comprehensive checklist for the flora of the islands. Data is formatted according to the Darwin Core standard (https://dwc.tdwg.org/terms). This dataset is available in the Global Biodiversity Information Facility, GBIF (Schrader 2020).

The dataset is also available in Suppl. material 5.

**Data set 5.** 

Column label	Column description
id	Unique ID for each occurrence record.
basisOfRecord	The specific nature of the data record. All samples were obtained from living specimens.
occurrenceID	Occurrence ID for GBIF: An identifier for the occurrence (as opposed to a particular digital record of the occurrence).
recordedBy	Names of collectors.
eventDate	Time frame of sampling.
islandGroup	The name of the island group in which the location occurs.
country	The name of the country in which the location occurs.
countryCode	The standard code for the country in which the location occurs (here ISO 3166-1 alpha-2).
decimalLatitude	The geographic latitude (in decimal degrees, using the spatial reference system given in geodeticDatum) of the geographic centre of a location.
decimalLongitude	The geographic longitude (in decimal degrees, using the spatial reference system given in geodeticDatum) of the geographic centre of a location.
geodeticDatum	The ellipsoid, geodetic datum, or spatial reference system (SRS) upon which the geographic coordinates given in decimalLatitude and decimalLongitude are based. Here: WGS84
coordinateUncertaintyInMeters	Indicator for the accuracy of the coordinate location, described as the radius of a circle around the stated point location in metres.
identificationQualifier	"cf." to express doubt about the species identification.
scientificName	The full scientific name of a taxon.
kingdom	The full scientific name of the kingdom in which the taxon is classified.
family	The full scientific name of the family in which the taxon is classified.
taxonRank	The taxonomic rank of the most specific name in the scientificName.

## Supplementary Material

B5A7D134-493D-5D8E-AE4D-8B84B82EC793 10.3897/BDJ.8.e55275.suppl1 Supplementary material 1 Island Data Data type Meta Data Brief description Data for 60 islands including island coordinates, geo-environmental variables, community summary statistics and number of sampling units. File: oo_444807.txt https://binary.pensoft.net/file/444807 Julian Schrader, Soetjipto Moeljono, Junus Tambing, Cornelia Sattler, Holger Kreft

14754988-0C59-524D-88B5-5E308FF9FC3A 10.3897/BDJ.8.e55275.suppl2 Supplementary material 2 Species data Data type Taxonomy Brief description Taxonomic list of all species found on the studied islands. File: oo_444808.txt https://binary.pensoft.net/file/444808 Julian Schrader, Soetjipto Moeljono, Junus Tambing, Cornelia Sattler, Holger Kreft

7CDA6A40-500B-538F-9C7C-F5461E6BE4EF 10.3897/BDJ.8.e55275.suppl3 Supplementary material 3 Community data Data type Species community data Brief description Community data for all individuals recorded on the studied islands, including occurrences in transects and plots, diameter at breast height and height. File: oo_444809.txt https://binary.pensoft.net/file/444809 Julian Schrader, Soetjipto Moeljono, Junus Tambing, Cornelia Sattler, Holger Kreft

A7DC1E53-F2CC-56E4-A305-F23CB60B8642 10.3897/BDJ.8.e55275.suppl4 Supplementary material 4 Plant functional trait data Data type Functional trait data Brief description Plant functional trait data for all species. File: oo_444810.txt https://binary.pensoft.net/file/444810 Julian Schrader, Soetjipto Moeljono, Junus Tambing, Cornelia Sattler, Holger Kreft

BFF4849C-13DF-52B3-AB64-F9D117931D09 10.3897/BDJ.8.e55275.suppl5 Supplementary material 5 Plant occurrences Data type Species occurrences Brief description Occurrence of all taxa that are identified at least to the level of genus. File: oo_419825.txt https://binary.pensoft.net/file/419825 Julian Schrader, Soetjipto Moeljono, Junus Tambing, Cornelia Sattler, Holger Kreft

## Figures and Tables

**Table 1.** Traits, ranges of trait values and numbers of species sampled. Two measures of tree height are provided. Height_max_ refers to the maximum height recorded for each species. Height_three_ refers to the mean height of the three tallest individuals of each species. For chlorophyll content, the concentration is in µm cm^-2^ (following Coste et al. 2010) and the measured SPAD units are provided. Trait values for all species can be found in Suppl. material 4.

**Trait**	**Unit**	**Range**	**No of species**
Fruit mass	g	0.01-20.03	44
Seed mass	g	0.00004-5.07	42
Height_max_	m	1.5-15.8	57
Height_three_	m	1.5-12.3	57
Wood density	g cm^-3^	0.29-0.99	53
Leaf mass per area (LMA)	g cm^-2^	0.52-2.6	56
Leaf area	cm^2^	1.78-126.66	56
Chlorophyll concentration	µm cm^-2^	19.45-114.55	52
Chlorophyll SPAD	SAPD unit	21.20-73.60	52
Leaf carbon	%	43.73-57.44	56
Leaf nitrogen	%	0.63-2.79	56
Leaf phosphorous	%	0.13-1.16	56
